# The Role of Inhalation Aromatherapy, Lavender and Peppermint in the Management of Perioperative Pain and Opioid Consumption Following Primary Unilateral Total Hip Arthroplasty: A Prospective, Randomized and Placebo-Controlled Study

**Published:** 2023-09-08

**Authors:** Jacques E Chelly, Brian Klatt, Michael O’Malley, Yram Groff, Jeremy Kearns, Sharad Khetarpal, Senthil Sadhasivam

**Affiliations:** 1 Department of Anesthesiology and Perioperative Medicine, University of Pittsburgh Medical Center, Pennsylvania, USA; 2 Department of Orthopaedic Surgery, University of Pittsburgh Medical Center, Pennsylvania, USA

**Keywords:** Aromatherapy, Acute pain, Opioids, Complementary medicine

## Abstract

**Introduction:**

Aromatherapy is claimed to be effective for the treatment of psychosocial disorders, but objective evidence of its effectiveness is still lacking. Psychosocial disorders have been demonstrated to increase postoperative pain and opioid consumption by up to 50%. This study was designed to assess the effectiveness of Aromatherapy in controlling postoperative pain and opioid in anxious patients.

**Methods:**

This prospective, randomized, placebo-controlled study was conducted on anxious patients who underwent primary unilateral total hip arthroplasty. After obtaining signed informed consent, each patient was asked to complete a PROMIS (Patient-Reported Outcomes Measurement Information System) anxiety survey. Patients whose T score were >57.2 were randomized to either an active treatment (Lavender Peppermint Elequil^®^ aromatab^®^) or a placebo Elequil^®^-aromatab^®^ treatment. Demographics, pain, opioid consumption, PONV, and psychosocial surveys were collected on Postoperative Day POD1, POD2, POD7 and POD30. At the time of discharge and on POD30, each patient was asked to complete a satisfaction questionnaire, and they were asked to complete an SF12 survey on POD30. Difference between means was assessed using absolute standardized mean differences.

**Results:**

Sixty patients were included in the intend-to-treat analysis. Use of lavender and peppermint was associated with a decrease of 26% in pain (POD7; 0.46), 33% in opioid consumption (POD2; 0.42), and 48% in acetaminophen consumption (POD7; 0.54) and a 78% decrease in PONV (POD2; 0.44). Psychosocial scores decreased following surgery (p=0.001). Overall satisfaction ratings at discharge were similar, as were functional recovery scores.

**Discussion:**

Our data provides evidence that in patients with preoperative anxiety, lavender and peppermint aromas decreases postoperative pain and opioid requirement compared to placebo. Additional research is required to conform our data.

**Conclusion:**

This randomized placebo control study provides evidence of the usefulness of inhalation of lavender and peppermint aromas in patients undergoing primary unilateral total hip arthroplasty.

## Introduction

Preoperative anxiety, depression, sleep disorders, and catastrophizing are common conditions that plague many surgical patients [1–10]. These conditions have been shown to negatively impact recovery and increase postoperative pain and opioids requirement by up to 50%. Evidence also supports the concept that the ability to control pre-operative anxiety, depression, sleep disorders, and catastrophizing prior to surgery eliminates the impacts that psychological factors may have on recovery [11].

The use of aromatherapy has been advocated for thousands of years due to its beneficial effects on psychological wellness [12]. However, in most cases, evidence has been based on anecdotal reports and/or non-validated questionnaires [13–16]. Recent reviews and meta-analyses have evaluated existing randomized clinical trials on aromatherapy and the treatment of pre-operative anxiety [17–19]. The results were non-conclusive, and the authors recommended “more vigorous placebo-controlled trials should be conducted to establish the efficacy of aromatherapy.” Wotman, et al. conducted a randomized, placebo-controlled study to assess the benefit of lavender inhalation treatment. Although the authors reported a significant decrease in preoperative anxiety in patients who received lavender inhalation treatment prior to surgery, in this study, patients estimated and rated their anxiety using a simple 1 to 10 analog scale (1=not anxious and 10=most anxious possible). The PROMIS questionnaire was developed and validated in recent years to objectively assess anxiety, depression and sleep disorders [20–21]. Furthermore, a validated questionnaire has been developed to assess catastrophizing behavior [22].

This study was designed to assess the role of inhalational aromatherapy on postoperative pain and opioid consumption in patients with preoperative anxiety using the PROMIS questionnaire. We selected lavender and peppermint aromas for testing because of their reported respective effects on anxiety and pain.

## Materials and Methods

### Study design and participants

**Figure 1:**
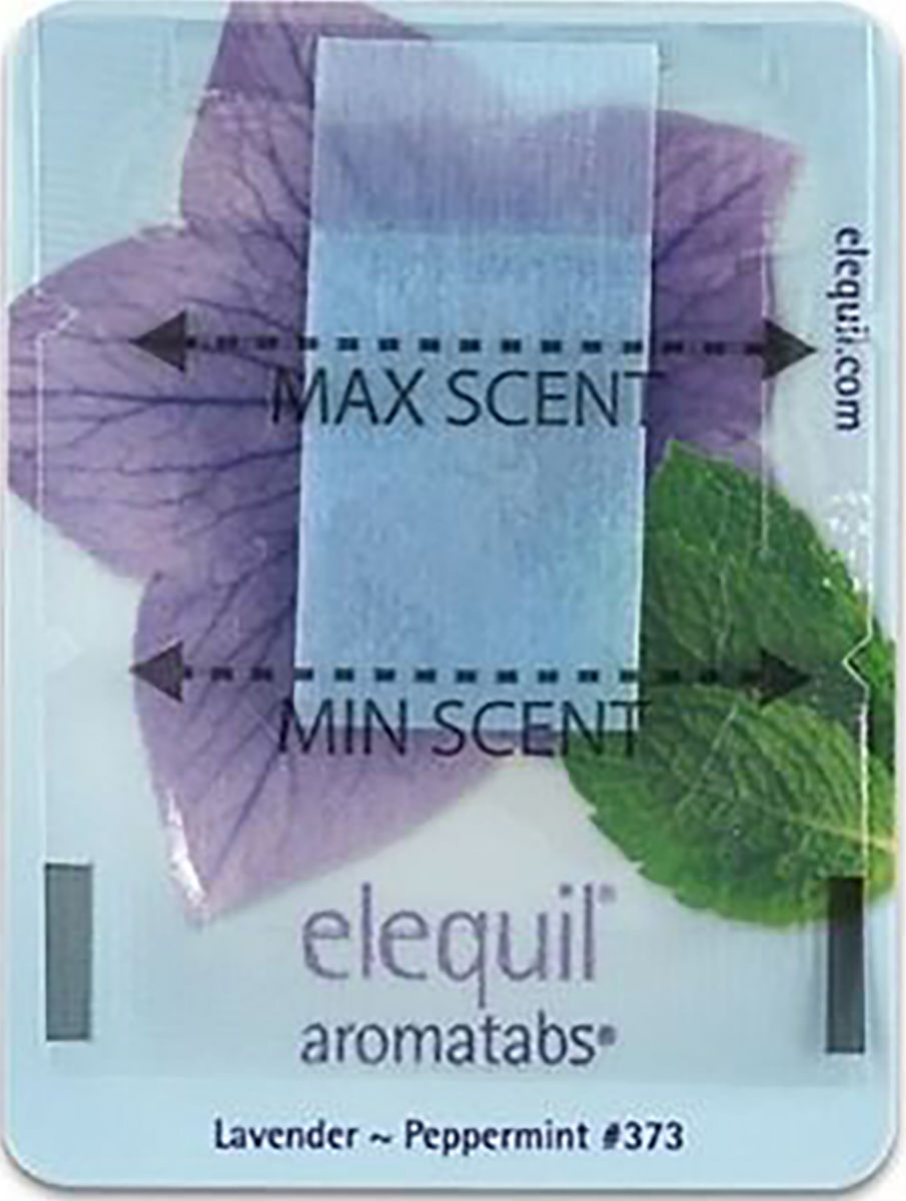
The self-adhesive Elequil^®^ aromatab^®^ patch was initially applied on each patient’s gown at chest level at least one hour prior to the patient’s transfer to the operating room. The Elequil^®^ Aromatab^®^ was open at the level of max aroma and left in place for 12 hrs. A total of six patches were used per patient. Both the treatment and placebo patches look identical.

The study was conducted as a single-center, prospective, randomized, placebo-controlled study at UPMC (University of Pittsburgh Medical Center) Shadyside Hospital. Institutional Review Board approval was obtained (STUDY20100091) and the trial was registered at www.clinicaltrials.gov (NCT04800744) before any eligible patients were recruited and consented. Each consented patient was asked to complete a PROMIS^®^ Emotional Anxiety Short Form 8a questionnaire. Inclusion criteria: Patients undergoing going primary unilateral Total Hip Arthroplasty (THA) or unilateral primary Total Knee Arthroplasty (TKA) and having signed an informed consent, 18 years of age or older, scoring ≥ 57.2 (T score) on the PROMIS^®^ emotional anxiety short form 8a questionnaire), and being opioid-naïve (use of less than 60 mg Oral Morphine Equivalent (OME) daily for the last 30 days). Exclusion criteria included pregnancy; chronic opioid use; uncontrolled clinical depression, anxiety, or catastrophizing; active alcoholism or drug abuse; severe chronic pain condition; fibromyalgia; almond allergy; participation in any other clinical trial; and being deemed unsuitable for the study at the discretion of the principal investigator. After obtaining signed informed consent, each patient was randomized to either an active or placebo treatment group. The active treatment was a Lavender-Peppermint (Self-adhesive Elequil^®^ aromatab^®^; Beekley Medical^®^, Bristolo, CT). Lavender and peppermint were chosen because of their proposed effectiveness on anxiety, sleep disturbance, pain, and Postoperative Nausea and Vomiting (PONV) (23–29). The placebo treatment consisted of a sweet Almond oil self-adhesive Elequil^®^ aromatab^®^ (Beekley Medical^®^, Bristol, CT). Both patches look identical (Figure 1). Each enrolled patient was also asked to complete a PROMIS^®^ Emotional Depression, a PROMIS^®^ Sleep Disturbance, pain catastrophizing survey prior to surgery (baseline).

The Elequil^®^ aromatab^®^ was placed on the patient gown prior to transport to the operating room. Each patient was also provided with additional five Lavender Peppermint Elequil^®^ aromatab^®^ or Almond Elequil^®^-aromatab^®^ and instructed to change their aromatab^®^ every 12 hours for the next two days after surgery. The first Elequil^®^ aromatab^®^ was kept on the patient during and after surgery. Surgery was performed under spinal anesthesia and propofol infusion for sedation. At discharge, each patient was provided with a three-day opioid prescription and instructed to contact the surgeon’s office for a refill if necessary. Pain (rated using a 0–10 numeric rating scale (0=no pain, and 10=the worst possible pain), opioid consumption (OME, mg), and PONV were evaluated on postoperative day (POD1, POD2, POD7 and POD30). Time to discharge from the recovery room and from the hospital was also recorded. Each patient was asked to rate their overall satisfaction at discharge and on POD30 and their overall pain treatment satisfaction (0=totally dissatisfied and 10=most satisfied) on POD30. Functional recovery was assessed using the SF-12 survey on POD30.

### Statistical analysis

Pain and opioid consumption were considered the primary outcomes. A power analysis indicated that at least 60 patients were required to demonstrate a difference of 20% between groups. An intent-to-treat analysis was conducted. Included in the analysis were two patients in the treatment group who stopped using the patch on POD1; one patient claimed the patch was inducing headache and nausea and the other patient indicated that they did not use the patch at home. Continuous data are presented as mean ± Standard Deviation (SD) and as percentages (%) for categorical variables. Differences between groups were compared using the Absolute Standardized Mean Differences (ASMDs). ASMDs of ≥ 0.2 were interpreted as showing an imbalance of means. Linear mixed models were used to assess the differences between baseline anxiety, depression, sleep disorder, and catastrophizing on POD 1, 2, 7 and 30 as well as between-group differences across days. Random effects were fit to control within person correlations across time. Interaction models were fit to test for differences between groups by timepoint. Alpha level was set at 0.05. ASMDs and mixed models were fit using R software (version 4.2.1, R Core Team, 2022). ASMDs and confidence intervals were fit using tidysmd and MBESS packages and mixed models were fit using the lmerTest package.

## Results

**Figure 2:**
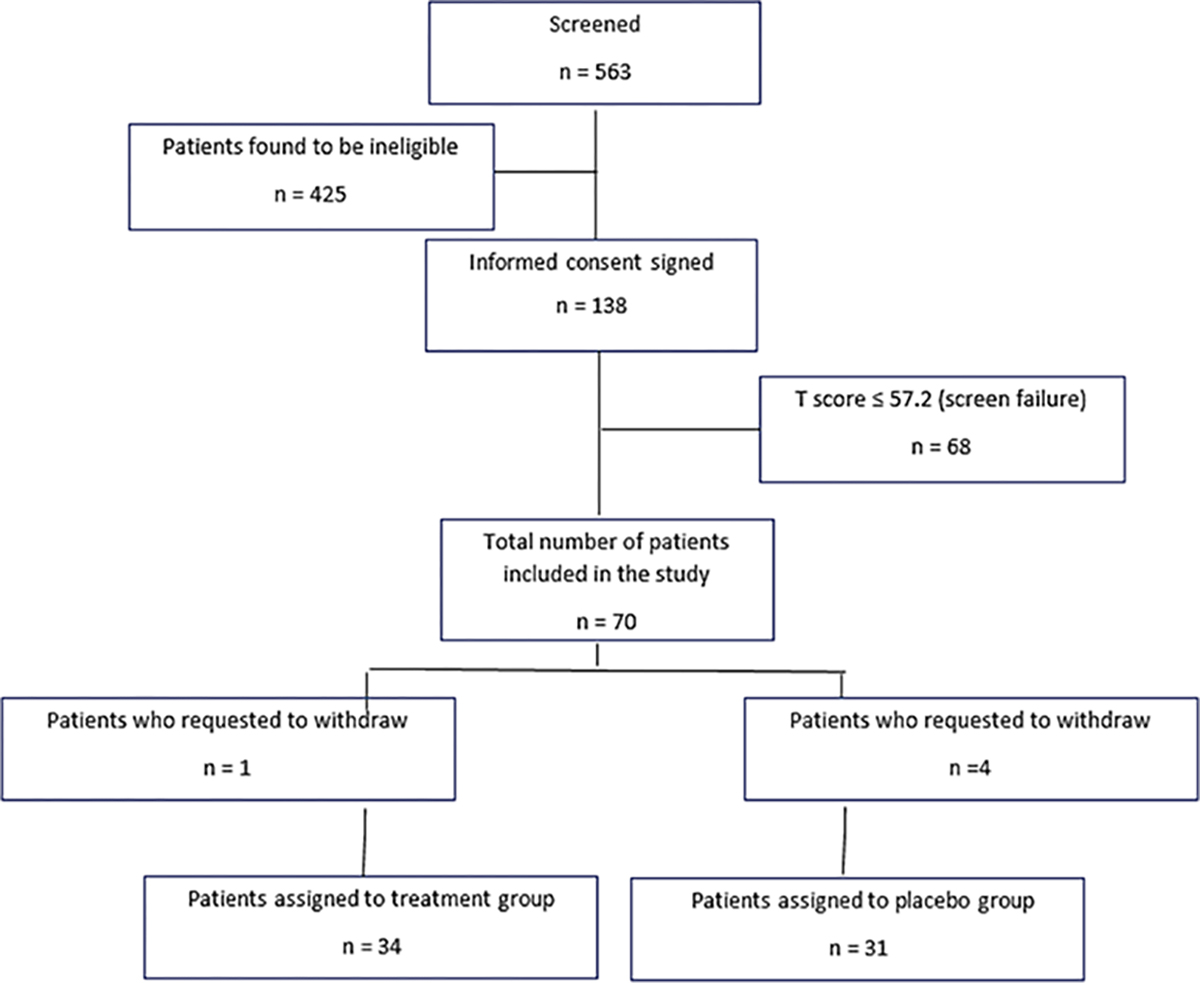
CONSORT diagram of study process.

A total 563 patients were screened, 279 were found eligible, and 138 signed an informed consent. Sixty-eight patients were not included because their PROMIS^®^ Emotional Anxiety Short Form −8a T scores were <57.2. (50.33 ± 5.79). Seventy patients with PROMIS^®^ Emotional Anxiety Short Form 8a T scores ≥ 57.2 were randomized, including five patients who withdrew from the study prior to discharge from the hospital (four patients in the placebo group and one patient in the treatment group who withdrew prior to transfer to the operating room because he/she “did not like the smell”). Figure 2 shows a CONSORT diagram of the study process.

Table 1 presents the patients’ demographic data (sex, race, weight, height, body mass index), types of surgery, PROMIS^®^ Emotional Anxiety scores, PROMIS^®^ Emotional Depression scores, PROMIS^®^ Sleep Disturbance scores, pain catastrophizing scores, and initial functional status prior to surgery. The PROMIS Emotional Distress-Anxiety and PROMIS Pain Interfering scores were found to be higher than those of the US population (50 ± 10), while the (PROMIS) Emotional Distress-Depression scores were not. Except for race, there was no evidence suggesting difference between means. White was the predominately represented racial group.

Compared to placebo, the use of the Lavender-Peppermint aromatab^®^ resulted in a maximum decrease of 26% in pain on POD7 (0.46*), 33% in opioid consumption on POD2 (0.42), and 48% in acetaminophen consumption on POD7 (0.54). Furthermore, the use of the Lavender-Peppermint Self-adhesive Elequil ^®^ aromatab^®^ resulted in a maximum decrease of 75% decrease in PONV scores on POD 2 (0.44*).

**Figure 3:**
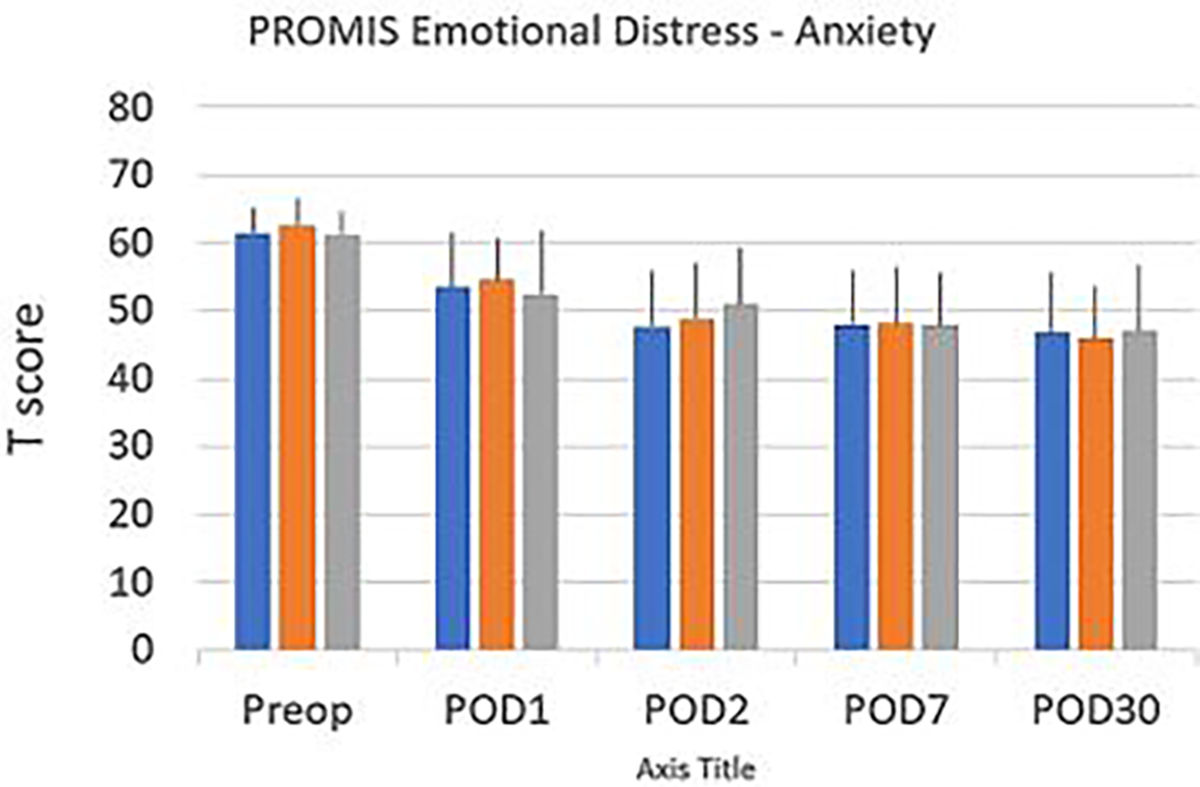
PROMIS^®^ Emotional Anxiety Short Form 8a T scores recorded prior to the patient’s transfer to the operating room (preop) on POD 1, POD 2, POD 7, and POD 30. No statistical differences were found between the overall scores or the scores recorded in the treatment and the placebo groups. Except for POD 1, no significant difference was reported between the treatment and the placebo group and the overall scores. Scores decrease significantly (p<0.001) on POD 1-POD30 compared to baseline (Preop). **Note:** (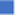) Over all (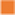) Placebo (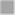) Treatment

**Figure 4:**
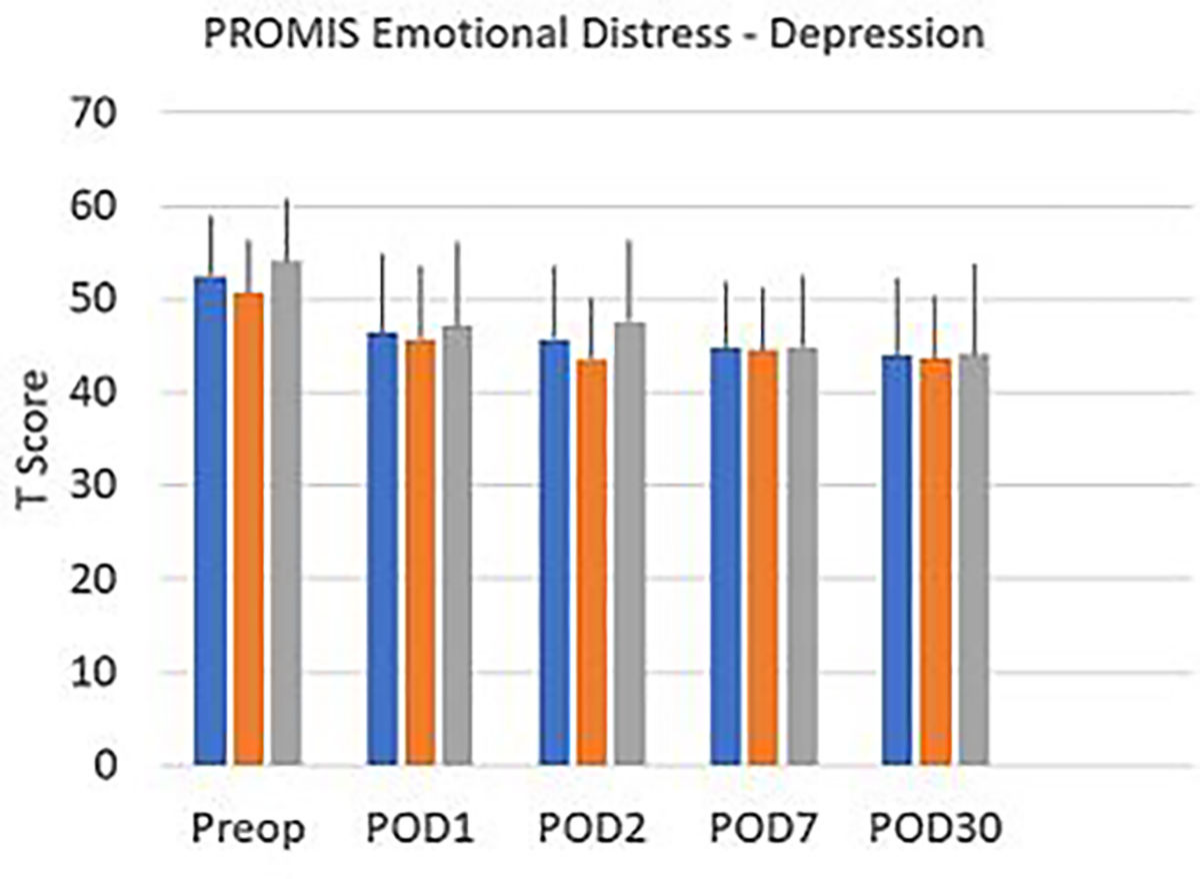
PROMIS^®^ Emotional Depression Short Form 8a T scores recorded prior to transfer to the operating room (preop) on POD 1, POD 2, POD 7, and POD 30. No statistical differences were found between the overall scores or the scores recorded in the treatment and the placebo groups. Data recorded prior to the transfer to the operating room, (baseline) on POD 1, POD 2, POD 7, and POD 30. No significant differences were reported between the treatment and placebo groups and the overall scores. Scores decrease significantly (p<0001) on POD 1-POD30 compared to baseline (Preop). **Note:** (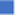) Over all (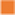) Placebo (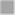) Treatment

**Figure 5:**
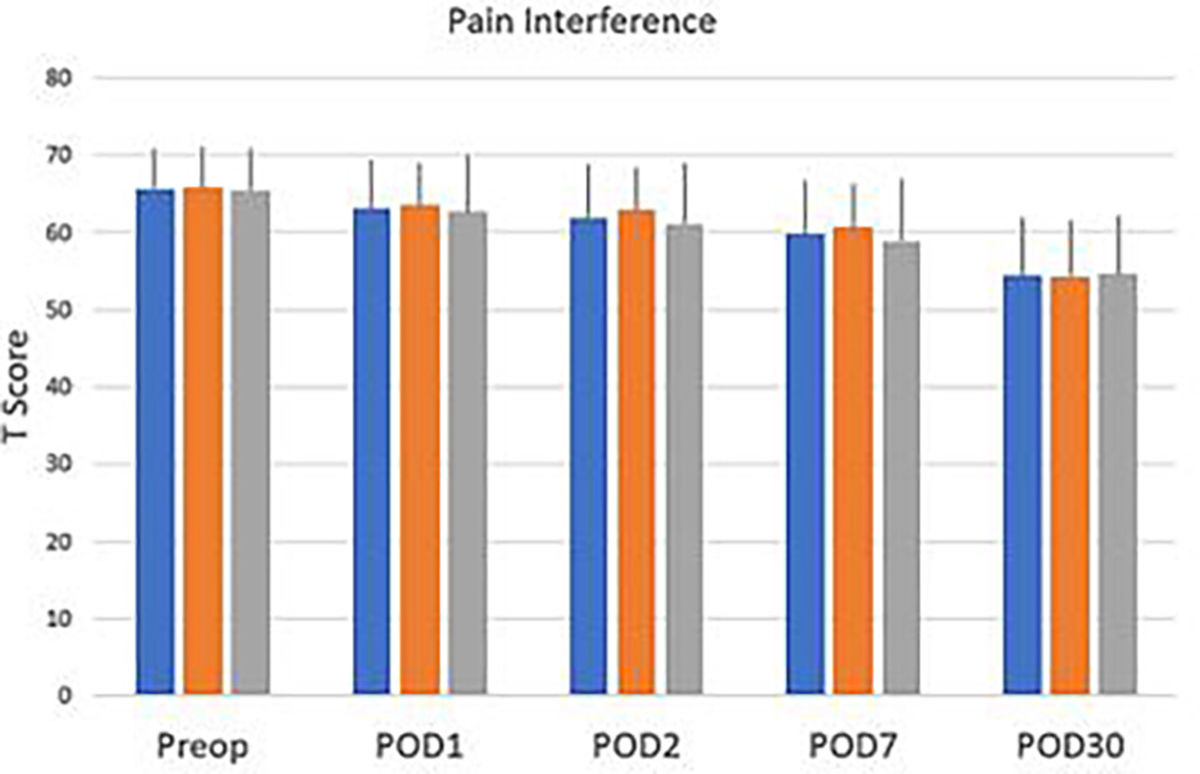
PROMIS^®^ Pain Interference Short Form 8a T scores recorded prior to patient transfer to the operating room (preop) on POD 1, POD2, POD 7, and POD 30. No statistical differences were found between the overall scores or the scores recorded in the treatment and the placebo groups. Data recorded prior to transfer to the operating room, (baseline) on POD 1, POD 2, POD 7, and POD 30. No significant difference was reported between the treatment and the placebo group and the overall scores. Scores decrease significantly (p<0.001) on POD 1-POD30 compared to baseline (Preop). **Note:** (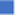) Over all (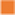) Placebo (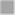) Treatment

**Figure 6:**
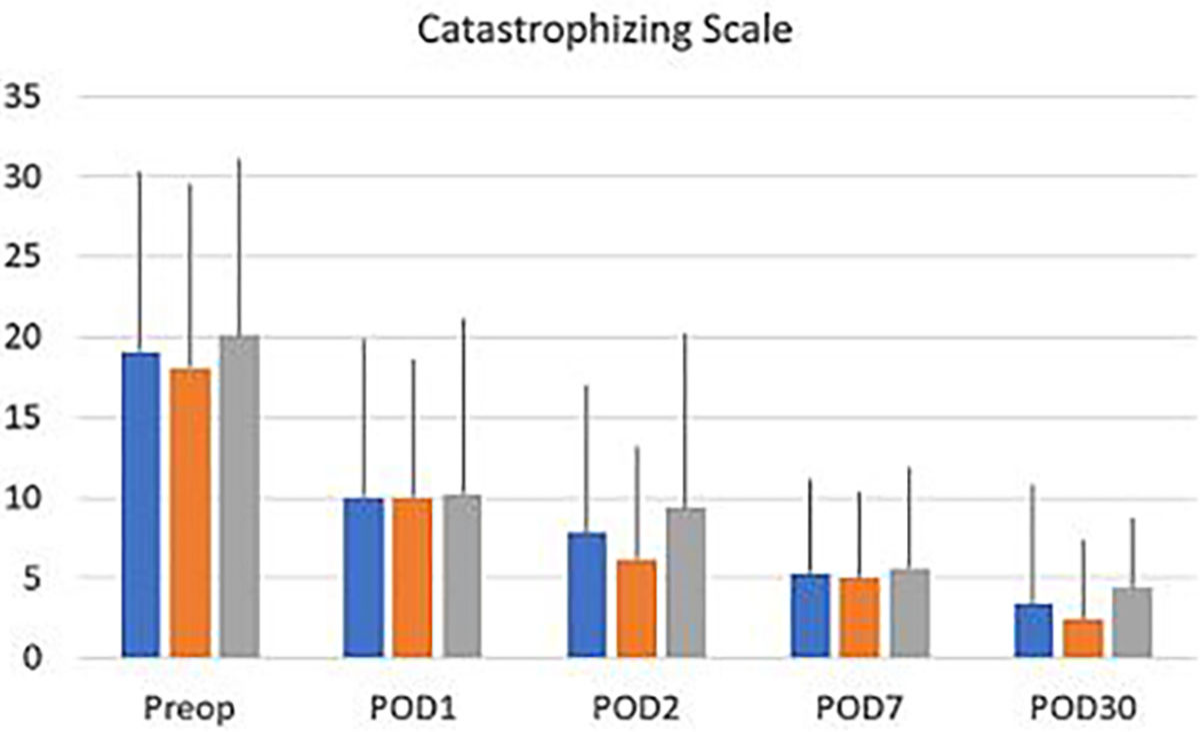
Catastrophizing scores recorded prior to transfer to the operating room (preop) on POD 1, POD 2, POD 7, and POD 30. No statistical differences were found between the overall scores and the scores recorded in the treatment and placebo groups. Data recorded prior to transfer to the operating room, (baseline) on POD 1, POD 2, POD 7, and POD 30. Except for POD 2, no significant difference was reported between the treatment and the placebo group. Scores decrease significantly (p<0.001) on POD 1-POD30 compared to baseline (Preop). **Note:** (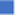) Over all (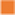) Placebo (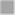) Treatment

Scores on the PROMIS emotional distress-anxiety, PROMIS emotional distress-depression, PROMIS sleep and pain interference and catastrophizing questionnaires were lower on POD1 compared to POD30. The magnitude of the drop varied according to the factor surveyed. In the treatment group, we recorded 12%, 21%, 22% and 26% decreases over time in PROMIS emotional distress-anxiety scores on POD 1, 2, 7 and 30, respectively Figure 3; 12%, 11%, 16%, and 17% decreases over time in PROMIS Emotional Distress-Depression scores on POD 1, 2, 7 and 30, respectively Figure 4; 4%, 7%, 10%, and 17% decreases over time in PROMIS Sleep and Pain Interference scores on POD 1, 2, 7 and 30, respectively Figure 5; and 49%, 53%, 72%, and 78% decreases over time in catastrophizing scores on POD 1, 2, 7 and 30, respectively Figure 6. Similar changes were recorded in the placebo group, suggesting that surgery rather than aromatherapy was the main contributor to the observed decrease. Table 2 presents outcomes between POD1-POD30 in the placebo and treatment groups.

Few adverse effects were reported. One patient in the placebo group removed the patch prior to surgery because they did not like the smell of the patch and one patient in the treatment group complained on POD1 that the patch was giving them a headache and nausea.

Overall satisfaction at discharge and on POD30 and overall pain treatment satisfaction on POD30 were similar in both groups (9.8 ± 0.4, 9.8 ± 0.4 and 9.6 ± 1.2, respectively in the placebo group vs. 9.8 ± 0.5, 9.8 ± 0.5 and 9.4 ± 1.5, respectively, in the treatment group (0.06)).

Functional recovery at POD30 was found to be similar in both groups (SF-12 Health Survey: PCS 37.1 ± 8.8 vs. 39.0 ± 8.2; (0.06) and MCS 56.6 ± 6.4 vs. 54.6 ± 1 (0.18)).

## Discussion

Our study provides evidence that the lavender-peppermint treatment started prior to surgery and continued for two days postoperatively via the use of an Self-adhesive Elequil ^®^ aromatab^®^ in patients undergoing primary hip replacement was associated with postoperative reduction in pain, opioid consumption, and PONV. Aromatherapy is based on administration of a single oil aroma over time or a combination of oil aromas, which in most cases requires the use of an inhaler. The use of inhalers for aromatherapy administration has limitations in the context of surgery due to the specific timing of administration. In most cases, the treatment take place prior to transfer to the operating room or after surgery in the recovery room [16–29]. The use of the aromatab^®^ allowed us to continuously administer the treatment starting prior to surgery and continuing after the surgery.

In this regard, the Self-adhesive Elequil ^®^ aromatab^®^ represents an innovative and practical mode of aromatherapy administration, requiring no inhaler and allowing a continuous delivery of a specific aroma or a combination of aromas over time. In our study, the aromatab^®^ was reported to be well accepted. This mode of administration is also inexpensive compared to the use of an inhaler. A unit cost around $2-$4, making the daily treatment less than $10/day.

Our study provides evidence that surgery is the main factor contributing to the high level of anxiety, depression, sleep disturbance, and catastrophizing recorded at baseline, since in both groups the scores decreased significantly following surgery.

## Conclusion

Although in our cohort anxiety, sleep disturbance, and catastrophizing scores were higher than those of the US population at baseline, depression scores were within the scope of those recorded in the US population. Furthermore, our data suggest that catastrophizing was the factor most affected by surgery since a 78% maximum reduction in catastrophizing scores was recorded at POD30 vs. a 26%, 17% and 17% reduction recorded for PROMIS anxiety, PROMIS depression, and PROMIS sleep and pain interference scores at POD30. Our randomized, placebo-controlled study demonstrated the positive effect of inhalational aromatherapy on anxiety, pain and opioid and acetaminophen requirement, applied via the continuous administration of lavender and peppermint for two days using the Self-adhesive Elequil ^®^ aromatab^®^ as a mode of administration. However, additional research is required to confirm these encouraging data and establish the mechanism of action of inhalation aromatherapy. One limitation of this study was the relatively limited number of patients included. Also, although the study design was robust, the power analysis was focused on pain and opioid requirement. The study was not powered for secondary variables.

## Figures and Tables

**Table 1:** Baseline characteristics by aromatherapy treatment arm.

Variable	All n=65	Control n=31	Treatment n=34	ASMD
**Sex**				0.15
Male n (%)	29 (44.6)	15 (48.4)	14 (41.2)	-
Female n (%)	36 (55.4)	16 (51.6)	20 (58.8)	-
Age (years) mean (SD)	61.6 (9.3)	61.1 (10.2)	62.0 (8.5)	0.15
**Race n (%)**				0.51*
White	61 (93.8)	30 (96.8)	31 (91.2)	-
African American	3 (4.6)	0 (0%)	3 (8.8)	-
Asian	1 (1.5)	1 (3.2)	0	-
Weight (kg) mean (SD)	84.4 (20.3)	85.1 (19.6)	83.8 (21.2)	0.06
Height (cm) mean (SD)	169.5 (14.0)	169.7 (16.6)	169.4 (11.2)	0.02
Body mass index (kg/m^2^) mean (SD)	28.8 (5.8)	28.8 (5.4)	29.0 (6.2)	0.05
PROMIS-Anxiety score mean (SD)	61.8 (3.8)	62.4 (4.1)	61.2 (3.4)	0.34* 0.4*
PROMIS-Sleep score mean (SD)	52.1 (6.6) 65.7 (5.3)	50.8(5.6) 65.9(5.2)	53.3 (7.2) 65.5 (5.5)	0.07
Pain catastrophizing score mean (SD)	19.2 (11)	18.4 (11.4)	20 (10.7)	0.15

**Note**: *: Absolute mean difference suggesting difference between control and treatment means

**Table 2:** Pain, Opioid consumption, PONV, PROMIS^®^ Emotional Anxiety Short Form 8a questionnaire (PROMIS Anxiety), PROMIS^®^ Emotional Depression Short Form 8a, (PROMIS Depression), PROMIS^®^ Sleep and Pain Interference questionnaires ( PROMIS Sleep disorder) and Pain Catastrophizing following surgery in the recovery room (PACU), POD1, POD2, POD7 and POD 30.

Control n=31		Treatment n=34	ASMD
**Pain**			
PACU	3.6 (3.2)	2.9 (2.9)	0.24*
POD1	5.4 (2.5)	4.6 (2.1)	0.32*
POD2	4.6 (2.2)	4.2 (2.2)	0.2*
POD7	4.2 (2.4)	3.1 (2.3)	0.46*
POD30	1.5 (2)	1.7 (1.6)	0.15
**Opioid consumption in OME (mg)**			
PACU	14.8 (11.2)	13.7 (2.9)	0.09
POD1	21.4 (14.7)	18.4 (14.7)	0.2*
POD2	17.2 (15.4)	11.6 (11.8)	0.42*
POD7	6.1 (7.5)	6.9 (10.9)	0.09
POD30	0.2 (0.9)	0.4 (1.6)	0.18
**Acetaminophen (mg)**			
PACU	209 (344)	196 (346)	0.04
POD1	1508 (1331)	1163 (874)	0.31*
POD2	1633 (1246)	1088 (871)	0.52*
POD7	1269 (1395	664 (201)	0.54*
POD30	405 (732)	514 (830)	0.14
**PONV Scores**			
PACU	0 (0.2)	0 (0)	0.26*
POD1	1.1 (2.3)	0.6 (1.3)	0.25*
POD2	0.8 (1.7)	0.2 (0.8)	0.44*
POD7	0.3 (0.8)	0.7 (1.4)	0.36*
POD30	0.2 (0.6)	0.1 (0.4)	0.24*
**PROMIS-Anxiety**			
POD0	62.4 (4.1)	61.2 (3.4)	0.34*
POD1	54.6 (6.2)	52.4 (9.3)	0.29
POD2	48.8 (8.2)	50.9 (8.4)	0.25*
POD7	48.1 (8.3)	47.8 (7.9)	0.04
POD30	45.8 (7.8)	47.0 (9.7)	0.14
**PROMIS-Depression**			
POD0	50.8 (5.6)	53.3 (7.2)	0.4*
POD1	47.7 (7.8)	47.0 (9.1)	0.15
POD2	43.5 (6.7)	47.6 (8.6)	0.55*
POD7	44.5 (6.7)	44.8 (7.7)	0.04
POD30	43.6 (6.8)	44.1 (9.7)	0.06
**PROMIS-Sleep**			
POD0	65.9 (5.2)	65.5 (5.5)	0.07
POD1	63.5 (5.4)	62.6 (7.4)	0.16
POD2	62.8 (5.5)	61.1 (8.0)	0.24*
POD7	60.6 (5.7)	58.8 (8.2)	0.26*
POD30	54.3 (7.2)	54.6 (7.6)	0.04
**Pain Catastrophizing**			
POD0	18.4 (11.4)	20 (10.7)	0.15
POD1	9.6 (8.7)	9.6 (11)	0.01
POD2	6 (7.0)	8.8 (10.8)	0.32*
POD7	4.8 (5.3)	5.2 (6.3)	0.08
POD30	2.3 (4.9)	4.2 (8.9)	0.26*

**Note**: *: Absolute mean difference suggesting difference between control and treatment means

## References

[1] Larach DB, Sahara MJ, Sanie SA, Moser SE, Urquhart AG, (2021) Patient factors associated with opioid consumption in the month following major surgery. Ann Surg 273:507–515. 31389832 10.1097/SLA.0000000000003509 PMC7068729

[2] Kuck K, Naik BI, Domino KB, Posner KL, Saager L, (2023) Multicenter perioperative outcomes group enhanced observation study investigator group for the multicenter perioperative outcomes group enhanced observation study collaborator group: prolonged opioid use and pain outcome and associated factors after surgery under general anesthesia: a prospective cohort association multicenter study. Anesthesiology 138:462–476. 36692360 10.1097/ALN.0000000000004510

[3] Rasouli MR, Menendez ME, Sayadipour A, Purtill JJ, Parvizi J (2016) Direct cost and complications associated with total joint arthroplasty in patients with preoperative anxiety and depression. J Arthroplasty 31:533–536. 26481408 10.1016/j.arth.2015.09.015

[4] Bedard NA, Pugely AJ, Westermann RW, Duchman KR, Glass NA, (2017) Opioid use after total knee arthroplasty: trends and risk factors for prolonged use. J Arthroplasty 32:2390–2394. 28413136 10.1016/j.arth.2017.03.014

[5] Riddle DL, Wade JB, Jiranek WA (2010) Major depression, generalized anxiety disorder, and panic disorder in patients scheduled for knee arthroplasty. J Arthroplasty 25:581–588. 19493643 10.1016/j.arth.2009.04.002

[6] Bistolfi A, Bettoni E, Aprato A, Milani P, Berchialla P, (2017) The presence and influence of mild depressive symptoms on post-operative pain perception following primary total knee arthroplasty. Knee Surg Sports, Traum, Arth 25:2792–2800. 10.1007/s00167-015-3737-y 26392343

[7] Pérez-Prieto D, Gil-González S, Pelfort X, Leal-Blanquet J, Puig-Verdié L,] (2014) Influence of depression on total knee arthroplasty outcomes. J Arthroplasty 29:44–47. 23702267 10.1016/j.arth.2013.04.030

[8] Feldman CH, Dong Y, Katz JN, Donnell-Fink LA, Losina E, (2015) Association between socioeconomic status and pain, function and pain catastrophizing at presentation for total knee arthroplasty. BMC Musculoskelet Disord 16:223–229. 25768862 10.1186/s12891-015-0475-8 PMC4329215

[9] Bonnin MP, Basiglini L, Archbold PHA (2011) What are the factors of residual pain after uncomplicated TKA? Knee Surgery, Sports Traumatology, Arthroscopy 19:1411–1417. 10.1007/s00167-011-1549-2 21598009

[10] Larsen BD, Laursen M, Simonsen O, Arendt-Nielsen L, Petersen KK (2021) The association between sleep quality, preoperative risk factors for chronic postoperative pain and postoperative pain intensity 12 months after knee and hip arthroplasty. Br J Pain 15:486–496. 34840796 10.1177/20494637211005803 PMC8611299

[11] Davin SA, Savage J, Thompson NR, Schuster A, Darnall BD (2022) Transforming standard of care for spine surgery: Integration of an online single-session behavioral pain management class for perioperative optimization. Front Pain Res (Lausanne) 3:852–256. 10.3389/fpain.2022.856252 PMC9118343 35599968

[12] Stea S, Beraudi A, De Pasquale D (2014) Essential oils for complementary treatment of surgical patients: state of the art. Evidence-based complementary and alternative medicine. Evid-based Complement Altern Med S1:1–5. 10.1155/2014/726341 PMC3953654 24707312

[13] Theunissen M, Peters ML, Bruce J, Gramke HF, Marcus MA (2012) Preoperative anxiety and catastrophizing: a systematic review and meta-analysis of the association with chronic postsurgical pain. Clin J Pain 28:819–841. 22760489 10.1097/AJP.0b013e31824549d6

[14] Shammas RL, Marks CE, Broadwater G, Le E, Glener AD, (2021) The Effect of lavender oil on erioperative pain, anxiety, depression, and sleep after microvascular breast reconstruction: A prospective, single-blinded, randomized, controlled trial. J Reconstr Microsurg 37:530–540. 33548936 10.1055/s-0041-1724465

[15] Jones T, Purdy M, Stewart EA, Cutshall SM, Hathcock MA, (2021) lavender aromatherapy to reduce anxiety during intrauterine insemination: A randomized controlled trial. Glob Adv Health Med 10:25–28. 10.1177/21649561211059074 PMC8606920 34820153

[16] Jaruzel CB, Gregoski M, Mueller M, Faircloth A, Kelechi T (2019) Aromatherapy for preoperative anxiety: A pilot study. JJ Perianesth Nurs 34:259–264. 10.1016/j.jopan.2018.05.007 30205934

[17] Choi J, Lee JA, Alimoradi Z, Lee MS (2018) Aromatherapy for the relief of symptoms in burn patients: A systematic review of randomized controlled trials. Burns 44:1395–1402. 29169701 10.1016/j.burns.2017.10.009

[18] Donelli D, Antonelli M, Bellinazzi C, Gensini GF, Firenzuoli F. (2019) Effects of lavender on anxiety: A systematic review and meta-analysis. Phytomedicine 65:153–199. 10.1016/j.phymed.2019.153099 31655395

[19] Kim M, Nam SK, Lee Y, Kang HJ (2021) Effects of lavender on anxiety, depression and physiologic parameters: Systematic Review and Meta-Analysis. Asian Nurs Res. 11:976–1317. 10.1016/j.anr.2021.11.001 34775136

[20] Wotman M, Levinger J, Leung L, Kallush A, Mauer E, (2017) The Efficacy of Lavender Aromatherapy in Reducing Preoperative Anxiety in Ambulatory Surgery Patients Undergoing Procedures in General Otolaryngology. Laryngoscope investigative otolaryngology 2:437–441. 29299520 10.1002/lio2.121 PMC5743169

[21] Cella D, Yount S, Rothrock N, Gershon R, Cook K, The Patient-Reported Outcomes Measurement Information System (PROMIS). Progress of an NIH roadmap cooperative group during its first two years. Med Care 2007 45: 93–111. 10.1097/01.mlr.0000258615.42478.55 PMC2829758 17443116

[22] Sullivan M, Bishop S, Pivik J (1995) The pain catastrophizing scale: Development and validation. Psychol Assess 7:524–532.

[23] Bozkurt P, Vural Ç (2019) Effect of lavender oil inhalation on reducing presurgical anxiety in orthognathic surgery patients. J Oral Maxillofac Surg 77:2466–2466. 10.1016/j.joms.2019.08.022 31574261

[24] Woelk H, Schläfke S (2010) A multi-center, double-blind, randomised study of the Lavender oil preparation Silexan in comparison to Lorazepam for generalized anxiety disorder. Phytomedicine 17:94–99. 19962288 10.1016/j.phymed.2009.10.006

[25] Trambert R, Kowalski MO, Wu B, Mehta N, Friedman P (2017) A Randomized controlled trial provides evidence to support aromatherapy to minimize anxiety in women undergoing breast biopsy. Worldviews Evid Based Nurs 14:394–402. 28395396 10.1111/wvn.12229

[26] Ozkaraman A, Dügüm Ö, Yılmaz HO, Yesilbalkan ÖU (2018) Clin Aromatherapy: The effect of lavender on anxiety and sleep quality in patients treated with chemotherapy. Clin J Oncol Nurs 22:203–10. 29547610 10.1188/18.CJON.203-210

[27] Darzi HB, Vahedian-Azimi A, Ghasemi S, Ebadi A, Sathyapalan T, (2020) The effect of aromatherapy with rose and lavender on anxiety, surgical site pain, and extubation time after open-heart surgery: A double-center randomized controlled trial. Phytother Res 34: 2675–2684. 32267031 10.1002/ptr.6698

[28] Briggs P, Hawrylack H, Mooney R (2016) Inhaled peppermint oil for postop nausea in patients undergoing cardiac surgery. Nursing 46:61–67. 10.1097/01.NURSE.0000482882.38607.5c 27333231

[29] Chumpitazi BP, Kearns GL, Shulman RJ (2018) Review article: the physiological effects and safety of peppermint oil and its efficacy in irritable bowel syndrome and other functional disorders. Aliment Pharmacol Ther 47:738–752. 29372567 10.1111/apt.14519 PMC5814329

